# Examination of the Efficacy and Cross-Reactivity of a Novel Polyclonal Antibody Targeting the Disintegrin Domain in SVMPs to Neutralize Snake Venom

**DOI:** 10.3390/toxins13040254

**Published:** 2021-03-31

**Authors:** Shelby S. Szteiter, Ilse N. Diego, Jonathan Ortegon, Eliana M. Salinas, Abcde Cirilo, Armando Reyes, Oscar Sanchez, Montamas Suntravat, Emelyn Salazar, Elda E. Sánchez, Jacob A. Galan

**Affiliations:** 1 National Natural Toxins Research Center (NNTRC), Texas A&M University-Kingsville, MSC 224, 975 West Avenue B, Kingsville, TX 78363, USA; SSzteiter@Rivieraisd.us (S.S.S.); n.diego@stridebio.com (I.N.D.); jonathan.d.ortegon@navy.mil (J.O.); eliana.m.salinas@students.tamuk.edu (E.M.S.); abcde.cirilo@students.tamuk.edu (A.C.); armando.reyes@students.tamuk.edu (A.R.); oscar.sanchez@tamuk.edu (O.S.); montamas.suntravat@tamuk.edu (M.S.); emelyn.salazarcastillo@tamuk.edu (E.S.); 2 Department of Chemistry, Texas A&M University-Kingsville, MSC 161, Kingsville, TX 78363, USA

**Keywords:** *C. atrox*, *C. o. helleri*, antivenom, disintegrin, SVMP PII/PIII, anti-disintegrin

## Abstract

Snake envenomation can result in hemorrhage, local necrosis, swelling, and if not treated properly can lead to adverse systemic effects such as coagulopathy, nephrotoxicity, neurotoxicity, and cardiotoxicity, which can result in death. As such, snake venom metalloproteinases (SVMPs) and disintegrins are two toxic components that contribute to hemorrhage and interfere with the hemostatic system. Administration of a commercial antivenom is the common antidote to treat snake envenomation, but the high-cost, lack of efficacy, side effects, and limited availability, necessitates the development of new strategies and approaches for therapeutic treatments. Herein, we describe the neutralization ability of anti-disintegrin polyclonal antibody on the activities of isolated disintegrins, P-II/P-III SVMPs, and crude venoms. Our results show disintegrin activity on platelet aggregation in whole blood and the migration of the SK-Mel-28 cells that can be neutralized with anti-disintegrin polyclonal antibody. We characterized a SVMP and found that anti-disintegrin was also able to inhibit its activity in an in vitro proteolytic assay. Moreover, we found that anti-disintegrin could neutralize the proteolytic and hemorrhagic activities from crude *Crotalus atrox* venom. Our results suggest that anti-disintegrin polyclonal antibodies have the potential for a targeted approach to neutralize SVMPs in the treatment of snakebite envenomations.

## 1. Introduction

Snake venom is the combination of many different types of enzymatic and non-enzymatic proteins and can cause toxicity upon envenomation. The predominant protein families found in *Crotalus atrox* (Western diamondback rattlesnake) venom are the L-amino acid oxidases, phospholipase A_2_s (PLA_2_s), serine proteinases (SVSPs), and metalloproteinases (SVMPs) [1]. In addition, nonenzymatic proteins are also found, which include cysteine-rich secretory proteins (CRiSPs), C-type lectins (CTL), and disintegrins. In the Viperidae family the most prominent are the SVMPs which can be as high as 70% of the total amount of protein [2]. Some of the toxins found in snake venom can lead to local tissue damage, swelling and edema, severe pain, functional disabilities, and can even cause death. On average, in the USA, it is estimated that there are 10,000 snakebites per year that require emergency treatment [3]. Of those bites, it has been determined that 4500 of the envenomations are from the Crotalidae family [3]. Several deadly rattlesnakes are found in California, but the one that seems to be the most predominant from the Viperidae family, is the *Crotalus oreganus helleri* (Southern Pacific rattlesnake), which can be found in the great central valley [4]. However, the *C. atrox*, accounts for most envenomations within northern Mexico and the United States [5]. Venom toxicity from both *C. atrox* and *C. o. helleri* can be hemotoxic, myotoxic, cytotoxic, and hemorrhagic. After envenomation, one can suffer from severe pain, vomiting, edema [6,7] and fluctuation of blood pressure [8].

Currently, antivenom is the only effective treatment for patients who get envenomated by snakes. If administrated on time and properly, one can survive a snakebite encounter. The only antivenoms available for treatment of Viperidae snakebites in North America are CroFab^®^ and Anavip^®^. CroFab^®^ is harvested from the blood of sheep that were immunized with 4 different North American snake venoms: *Crotalus scutulatus* (Mojave rattlesnake), *Agkistrodon piscivorus* (cottonmouth or water moccasin), *Crotalus adamanteus* (Eastern diamondback rattlesnake), and *Crotalus atrox* (Western diamondback rattlesnake) (www.crofab.com (accessed on 30 March 2021)). Moreover, CroFab^®^ contains Fab fragments from immunoglobulin G (IgG) that bind to snake venom toxins and neutralize their activities. Anavip^®^ is an equine-derived antivenom, contains F(ab’)_2_, and uses *Bothrops asper* (Fer-de-lance) and *Crotalus durissus* (South American rattlesnake) in the immunization protocols.

Although antivenoms are the only effective treatment for a snakebite envenoming, they come with profound limitations. Most antivenoms are made of antigen binding fragments F(ab’), F(ab’)_2_ or of whole immunoglobulins (IgGs) produced towards snake venom by the immunization of animals [9,10,11], however, approximately 70% of these immunoglobulins are not specifically directed towards the snake venom toxins [10,12] but are directed against the parasites and microorganisms found in immunized animals, which they had prior to venom immunization [13]. Another drawback for antivenoms is that they can cause reactions such as anaphylactic shock and serum sickness. These types of side effects can be caused by the whole IgG resulting in adverse reactions within the first 24 h of antivenom administration [14,15,16]. Moreover, the costs per vial of antivenom is another major problem, as each vial of antivenom costs approximately $2300 (more in some places), and an initial dose per envenomation starts between 4–6 vials ($9200–13,800 USD). Indeed, the average amount of vials used to effectively neutralize a snake envenomation is between 23–46 vials ($52,900–105,800 USD). Therefore, developing novel antivenom therapies to effectively neutralize snake venom while reducing cost is warranted.

In this work, we sought to determine the neutralizing efficacy of novel polyclonal disintegrin antibodies on the activity of SVMP PII/PIII. We hypothesize that the allosteric inhibition of a metalloproteinase via its disintegrin domain can be an effective strategy to neutralize snake venom toxicity and possibly its lethality. This characterization was carried out using native a disintegrins from *C. atrox*, snake venom metalloproteinases from *C. o. helleri*, and testing for the neutralizing ability of an anti-disintegrin against the platelet aggregation activity of disintegrins, and activity of SVMPs, respectively. We further tested, in vitro, the neutralization cross-reactivity of the anti-disintegrin against crude venom in 11 snakes from *C. atrox*, *C. o helleri* and *C. s. scutulatus.* Lastly, using anti-disintegrin, we neutralized crude *C. atrox* venom in vivo.

## 2. Results

### 2.1. Rationale and Scheme for Disintegrin Neutralization

**Figure 1:**
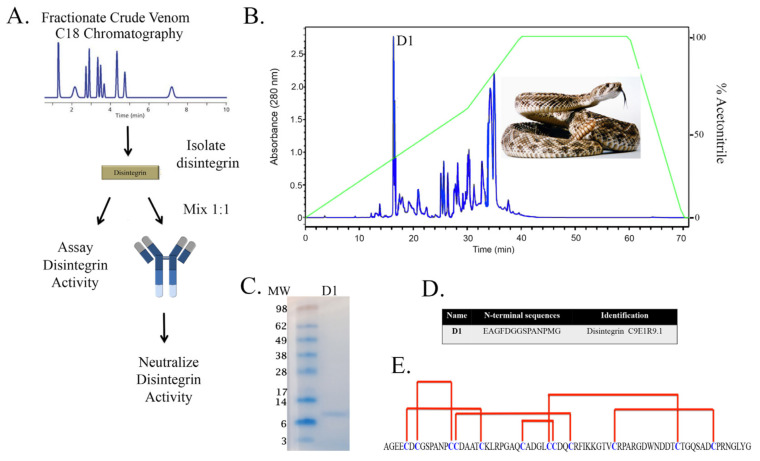
Neutralization strategy for the characterization of anti-disintegrin against disintegrins. (**A**) Schematic representation of approach to characterize and neutralize disintegrins with anti-disintegrin. (**B**) Isolation of disintegrin D1. A total of 200 µL of venom solution (30 mg/mL) was injected into a Reverse Phase C18 column as described in the Materials and Methods. (**C**) SDS-PAGE analysis of the D1 peak collected by reverse phase HPLC. A total of 3 µg of D1 was run on 4–12% Bis-Tris Gel under non-reducing conditions at 100 V for 95 min. The gel was stained with SimplyBlue Safestain. lanes 1: SeeBlue Plus2 Markers, lane 2: D1. (**D**) N-terminal sequence of D1. The identity of the primary sequence was determined using Basic Local Alignment Search Tool (BLAST http://blast.ncbi.nlm.nih.gov/Blast.cgi (accessed on 29 March 2021)). (**E**) Amino acid sequence of *C. atrox*, *C. horridus*, and *C. s. scutulatus* disintegrins. Intra molecular cysteine linkages are proposed from Juarez et al. 2008 [18].

We used a multi-step systematic approach to characterize and neutralize a disintegrin, PII/PIII SVMP and crude venom to determine the potential of our novel antibody. We previously created and developed a polyclonal antibody (anti-disintegrin) from the mojastin disintegrin, and have used this antibody to characterize Mohave rattlesnake type A/B and other various hybrids [17]. We next sought to determine if this antibody could neutralize whole snake venom. A detailed scheme illustrates the first step, which requires the purification, activity assay, and neutralization of a native disintegrin (Figure 1A). We fractionated the venom of *C. atrox* using C18 reverse phase chromatography. At 15 min, a highly purified fraction confirmed to be a disintegrin D1 (Figure 1B). Under non-reducing conditions, D1 displayed one band between 6 and 8 kDa (Figure 1C) and this was further confirmed by N-terminal sequencing (Figure 1D). The complete sequence of the disintegrin, crotastatin, showed a high degree of cysteine-rich residues required for intramolecular disulfide bridge and the RGD binding motif. Based on our lab’s extensive analysis on disintegrins from *Crotalus* snakes, the native disintegrins isolated from *C. atrox*, *C. horridus* and *C. s. scutulatus* are 100 % identical (Figure 1E).

**Figure 2:**
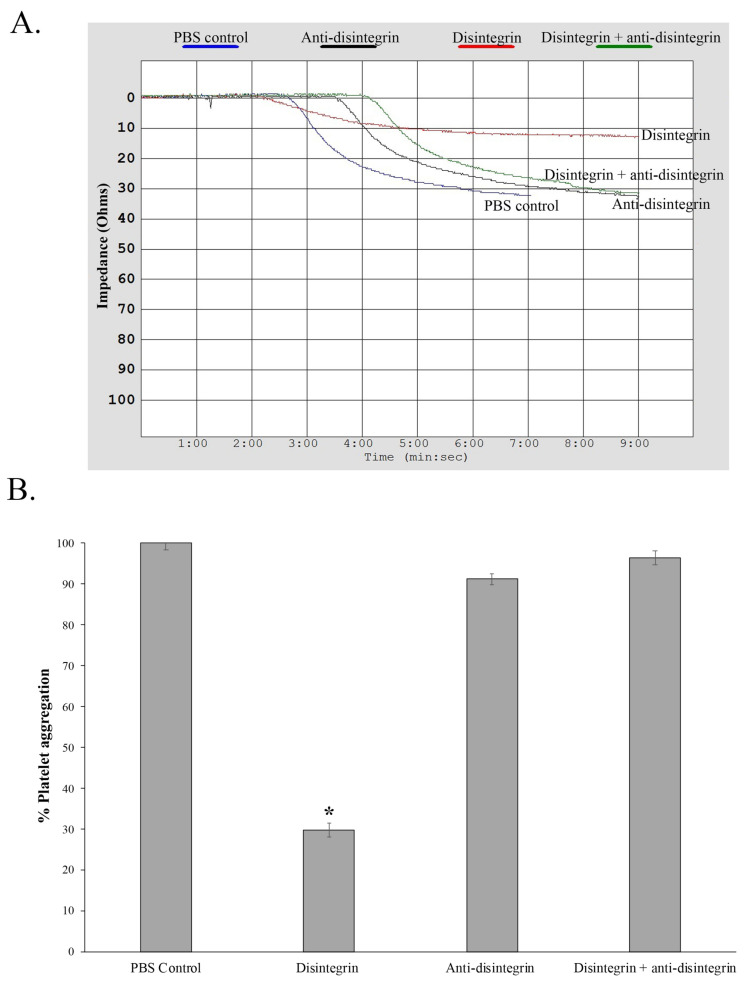
Characterization and neutralization of D1 on platelet aggregation. Neutralization of disintegrin-induced platelet aggregation inhibition by anti-disintegrin using human whole blood. (**A**) Illustrative aggregation curves recorded by the Chronolog aggregometer in whole blood. Chronolog aggregometer was used to measure ADP-induced platelet aggregation by impedance. (**B**) Neutralization of anti-platelet aggregation activity of disintegrin isolated from *C. atrox* by anti-disintegrin. A total of 10 μL of disintegrin/anti-disintegrin mixture was added to whole blood and incubated 2 min at 37 °C prior to adding 10 μM of ADP. The error bars represent the standard deviation from two independent experiments with *n* = 3. * Indicates significant differences between the PBS control and the disintegrin control (*p* < 0.05).

Since disintegrins are well known antagonist of platelet aggregation, we tested disintegrin D1 activity by quantifying the inhibition of platelet aggregation in whole blood using a platelet aggregometer. The phosphate buffer saline (PBS) control displayed platelet aggregation impedance of 14 ohms, indicating normal aggregation. Native disintegrin D1 was capable of inhibiting platelet aggregation ~70% with an impedance reading of 4 ohms. D1 was then incubated with anti-disintegrin for 30 min at 37 °C and was tested for anti-aggregation activity. Intriguingly, restored impedance of 14 ohms (100% restored) was observed, suggesting the anti-disintegrin could neutralize native disintegrin (Figure 2A,B).

**Figure 3:**
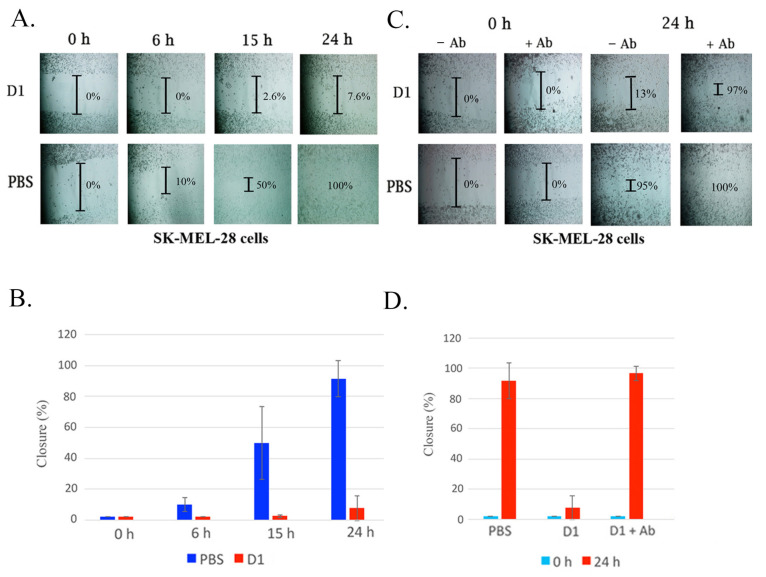
Characterization and neutralization of D1 on cell migration using wound healing analysis. (**A**) A confluent monolayer of cells was maintained in the medium with an insert generating a wound. Inhibition of SK-MEL-28 cell migration was measured when the disintegrin was added to tissue culture media. SK-MEL-28 cell lines were incubated with D1 (0.5 mg/mL) for 24 h. Cells were allowed to migrate and were monitored at 0 h, 6 h, 15 h, and 24 h at 37 °C. Untreated cells with PBS were used as a control. Results are expressed as cell closure percentage relative to the PBS control, $\bar{X}$ ± SD (*n* = 3). The extent of wound closure was quantified by multiple measurements of the width of the scratched area. (**B**) Quantification of inhibition of cell migration by snake venom a disintegrins. (**C**) Wound healing analysis using anti-disintegrin to neutralize the inhibition of cell migration of SK-MEL-28 cells was measured when the disintegrin was mixed with anti-disintegrin antibody and added to tissue culture media. A confluent monolayer of cells was maintained in the medium with an insert to leave a wound. The cultures were allowed to migrate for 24 h at 37 °C in the presence of disintegrin and disintegrins mixed with anti-disintegrin antibody. D1 (0.5 mg/mL) was pre-incubated with anti-disintegrin for 30 min. The samples were then added to SK-MEL-28 cells and incubated for 24 h. The extent of wound closure was quantified by multiple measurements as described in (A). (**D**) Quantification of inhibition of disintegrin activity by anti-disintegrin. Results are expressed as cell closure percentage relative to the PBS control, $\bar{X}$ ± SD (*n* = 3).

Native disintegrin D1 was tested for inhibition of cell migration activity using the wound healing assay. To determine the disintegrin activity in a wound healing assay, a “scratch” was formed on SK-MEL-28 cells, and the inhibitory activity of D1 on cell migration was monitored by collecting cell images using microscopy for 24 h. As expected, cells incubated with D1 resulted in 7.6% cell migration (~95% inhibition), compared to PBS control which was 100% (Figure 3A,B). Next, we tested our antibody for neutralization on disintegrin activity. The disintegrin was pre-incubated with the antibody for 30 min and then added to the cells. The cells were monitored for 24 h in a wound healing assay. At 24 h, the cells that contained the disintegrin/anti-disintegrin mixture had a 97% closure when compared to the cells that only contained disintegrin D1 (13%) (Figure 3C,D). No effects on cell proliferation were found for the disintegrin D1 (Supplemental Figure S1). These data show that our antibody can not only bind and detect disintegrins but could neutralize a native disintegrin as well. However, further analysis is warranted to characterize the precise mechanism of disintegrin/antibody neutralization.

### 2.2. Rationale and Scheme for Partially Purified Metalloprotease Neutralization

**Figure 4:**
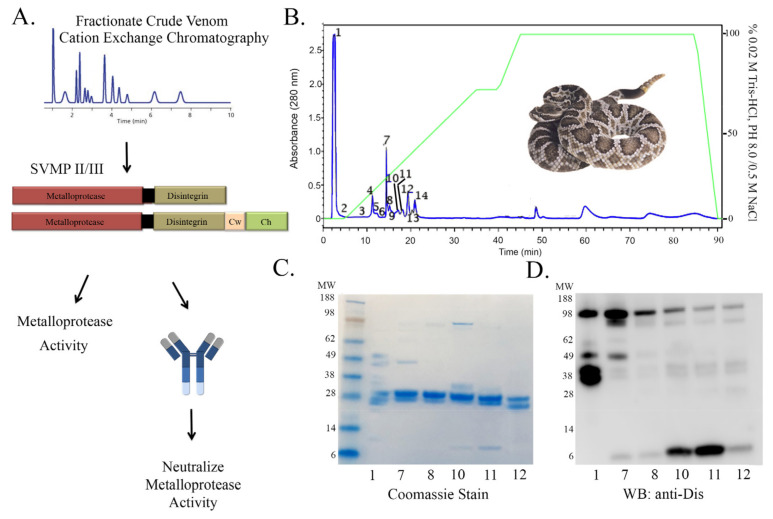
Neutralization Strategy for the characterization of anti-disintegrin against SVMPs. (**A**) Schematic representation of approach to characterize and neutralize SVMPs with anti-disintegrin. (**B**) Isolation of SVMPs. Two hundred microliters (30 mg/mL) of *C. o. helleri* venom was injected in a Waters Protein-Pak™ HPLC column (7.5 × 75 mm). The fractions were separated with 0.02 M trishydroxyaminomethane, 0.5 M NaCl, pH 8.0 as described in the Materials and Methods. (**C**) Purification of SVMPs SDS-PAGE analysis of *C. o. helleri* venom fractions collected by cationic exchange HPLC. A total of 5 µg of each fraction were separated on 4–12% Bis-Tris Gel under non-reducing conditions at 100 V for 95 min. The gel was stained with SimplyBlue Safestain. SeeBlue Plus2 Markers, Fractions 1, 7, 8, 10, 11, 12. (**D**) Immunoblot of cation exchange fractions probed with anti-disintegrin. Cation exchange fractions separated on SDS-PAGE were transferred to an Immobilon^®^ membrane (Millipore) using BioRad Trans-Blot SD Semi-Dry Transfer Cell at 100 V for 1 h and then left overnight. SeeBlue Plus2 Markers, Fractions 1, 7, 8, 10, 11, 12.

We next sought to characterize a SVMP PII/PIII by purifying an active enzyme, testing its activity and the ability of our antibody to neutralize its proteolytic activity (Figure 4A). We used the venom from a *C. o. helleri* based on previous and extensive knowledge of the venom composition and fraction activity. To fractionate an active SVMP, we used cation exchange chromatography (Figure 4B). Fourteen fractions were collected and the most abundant peaks were run on SDS-PAGE. Some fractions were excluded from further analysis as no protein species greater than 3 kDa was observed indicating smaller peptides containing aromatic amino acids. The SDS-PAGE indicated nearly purified proteins and a dominant band that appeared between 28 and 38 kDa (Figure 4C). To confirm the existence of a SVMP, we used our antibody to analyze the fractionated venom using immunoblot techniques. Immunoblot analysis showed many immunoreactive bands but not at 28 and 38 kDa, with the exception of fraction 1. Our data suggest that lanes 2–4 (Figure 4D), (fractions 1, 7, and 8) have a higher abundance of SVMPs at 98 kDa. Interestingly, lanes 4 and 5 (fractions 10 and 11) revealed strong immunoreactive bands for disintegrins at 7 kDa (Figure 4D).

**Figure 5:**
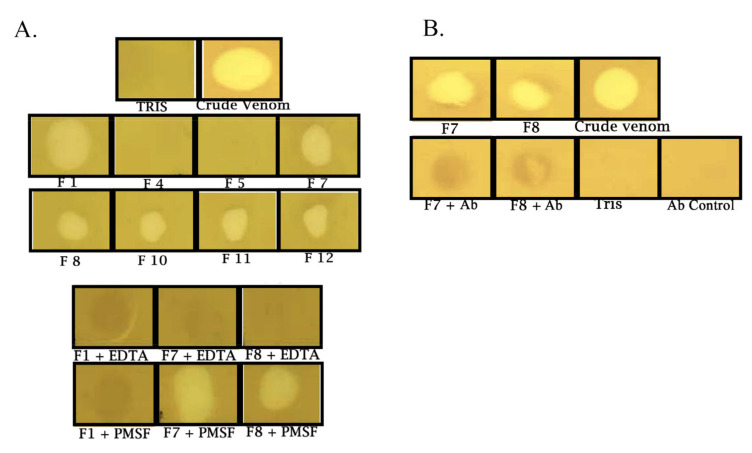
Characterization of SVMP proteolytic activity and neutralization using anti-disintegrin. (**A**) Gelatinase activity of different *C. o. helleri* venom fractions. Twenty microliters of each fraction were placed on the gelatin film as described in the Materials and Methods. The proteolytic activity was measured by the clearing of a 10 mm spot on the film. Determination of SVMPs and SVSPs using inhibitors. Fractions 1, 7 and 8 were first incubated with inhibitors, 2 mM EDTA or 2 mM PMSF for 30 min, and 20 µL of sample were applied onto the gelatin film. (**B**) Neutralization of gelatinase activity of SVMPs using anti-disintegrin. Fractions 7 and 8 were first incubated with anti-disintegrin for 30 min and then 20 µL of each fraction were applied on the gelatin film and the proteolytic activity was measured. Crude venom and anti-disintegrin (Ab) were used as positive and negative controls, respectively.

The most abundant fractions were tested on a proteolytic activity assay, using gelatin as a substrate. We found fractions 1 and 7–12 to have proteolytic activity on gelatin by a 10 mm clearing on the gelatin film. Fractions 4 and 5 did not have any gelatinase activity, as no detectable clearing appeared on the film (Figure 5A). Serine proteases can also have gelatinase activity; therefore, we needed to validate and confirm the isolation of a metalloproteinase. Using the anti-disintegrin immunoblot (Figure 3D) indicating high molecular weight bands for PII-SVMPs, we postulated that fractions 1, 7 and 8 contained partially purified metalloproteinases. We preincubated fractions 1, 7 and 8 with 2 mM ethylenediaminetetraacetic acid (EDTA) or 2 mM phenylmethylsulfonyl fluoride (PMSF), a metalloprotease inhibitor and serine protease inhibitor, respectively, for 1 h at room temperature. As predicted, fractions 7 and 8 were inhibited with EDTA but not with PMSF. Fraction 1 was inhibited by EDTA and PMSF (Figure 5A). We also confirmed these were partially purified SVMPs by N-terminal sequencing by excising the bands at molecular weight 49 kDa and 98 kDa for fractions 7 and 8, respectively (Supplemental Figure S2A). Anti-disintegrin was used to test if both fractions 7 and 8 containing metalloproteinase activity on a gelatinase assay could be neutralized. Both fractions were incubated with anti-disintegrin for 30 min prior to being applied to the gelatin film. Indeed, no clearing appeared in the fractions that had been incubated with anti-disintegrin, suggesting that the metalloprotease activities of fractions 7 and 8 were neutralized in vitro (Figure 5B).

### 2.3. Rationale and Scheme for Crude Venom Neutralization

**Figure 6:**
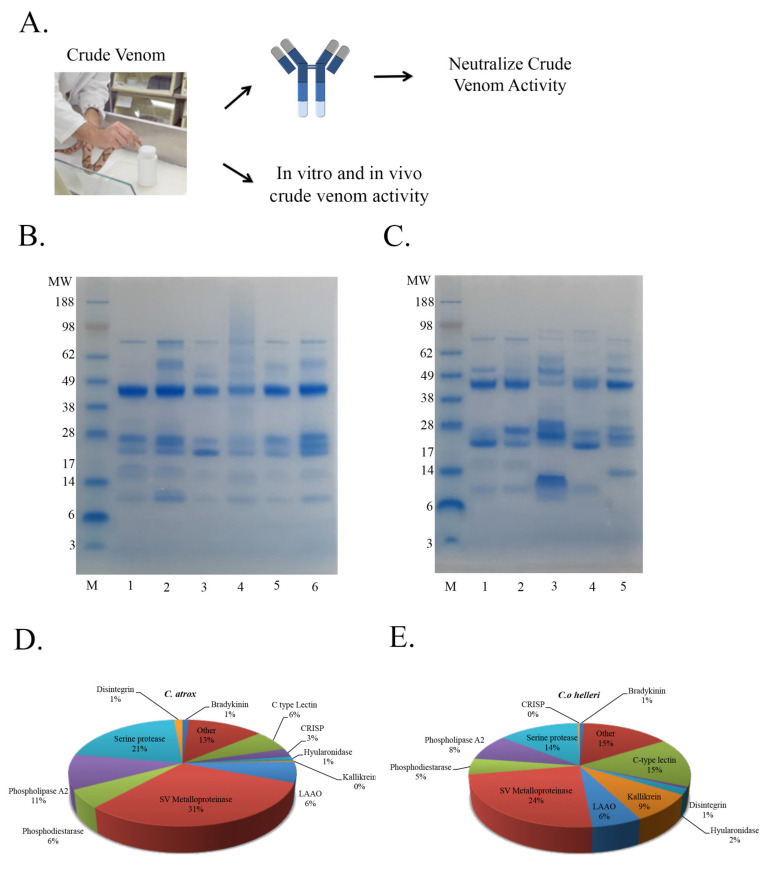
Strategy and characterization of snake venom. (**A**) Schematic representation of venom extraction and neutralization strategy of crude venom using anti-disintegrin. (**B**) SDS-PAGE analysis of crude venoms from different individual snakes tested using anti-disintegrin. A total of 10 μg of samples was run on a 4–12% Bis-Tris (MES) Gel (Novex^®^) at 100 V for 95 min. M: Seeblue^®^ Plus 2 prestained standard (1X), lanes 1: *C. atrox* (Luke), Lane 2: *C. atrox* (977), Lane 3: *C. atrox* (53), Lane 4: *C. atrox* (54), Lane 5: *C. atrox* (313), Lane 6: *C. atrox* (312). (**C**) SDS-PAGE analysis of crude venoms tested using anti-disintegrin M: Seeblue^®^ Plus 2 prestained standard (1X), lanes 1: *C. atrox* (68), Lane 2: *C. atrox* (143), Lane 3: *C. o. helleri* (792), Lane 4: *C. o. helleri* (680), Lane 5: *C. s. scutulatus* (931). (**D**) LC-MS/MS proteomic analysis of crude *C. atrox* venom. (**E**) LC-MS/MS proteomic analysis of crude *C. o. helleri* venom.

Inasmuch as the activity of a partially purified PII/PIII SVMP was inhibited, we next sought to assess the neutralization of crude venom using our anti-disintegrin (Figure 6A). Venom composition profiling using SDS-PAGE (Figure 6B,C), HPLC and immunoblotting using anti-disintegrin (Supplemental Figure S2B–D) reveals the protein complexity of the venoms used in our study. Proteomic analysis of *C. o. helleri* and *C. atrox* venom composition revealed the high abundances of SVMPs (Figure 6D,E), suggesting that both venoms had the potential to be neutralized by anti-disintegrin.

**Figure 7:**
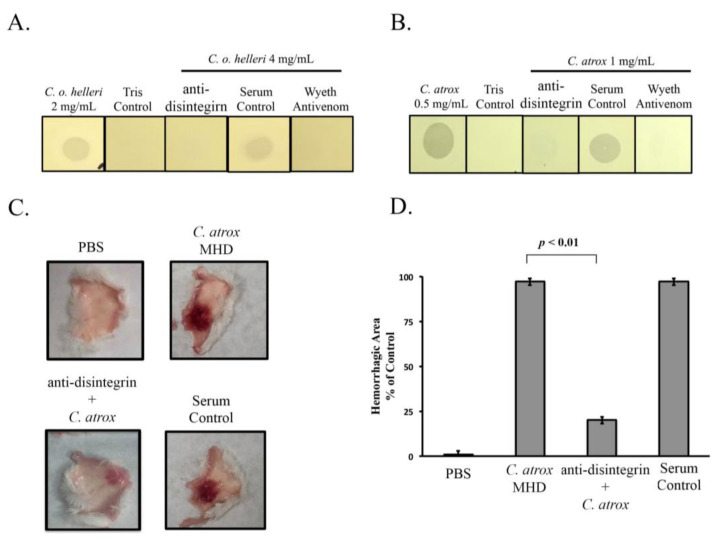
Neutralization of snake venom using anti-disintegrin. (**A**) Proteolytic neutralization of *C. o. helleri* venom using anti-disintegrin. A 2MGD of *C. o. helleri* venom was incubated for 30 min with serum control, anti-disintegrin or Antivenin Polyvalent (Wyeth) antivenom at equal volumes and then 20 µL of sample were applied onto the gelatin film and incubated for 4 h. (**B**) Proteolytic neutralization of *C. atrox* venom using anti-disintegrin. A 2MGD of *C. atrox* venom was incubated for 30 min with serum control, anti-disintegrin or Antivenin Polyvalent (Wyeth) antivenom at equal volumes and then 20 µL of sample were applied onto the gelatin film and incubated for 4 h. (**C**) Neutralization of hemorrhagic activity by anti-disintegrin. A total of 0.1 mL of MHD of *C. atrox* venom or venom/anti-disintegrin mixture was injected subdermally into the abdomen of BALB/c mice. After 24 h, the mice were sacrificed, and the skin removed for measurement of hemorrhagic area. The controls consisted of PBS, serum control, and MHD. An MHD contained 2.5 µg of venom protein capable of producing a 10 mm hemorrhagic spot. (**D**) Hemorrhagic activity was quantified by densitometry and is expressed as the hemorrhagic area percentage relative to the PBS control, $\bar{X}$ ± SD (*n* = 3).

We first used crude *C. o. helleri* and *C. atrox* venoms to test for anti-disintegrin neutralization. The minimal gelatinase dose (MGD) for each venom was 0.25 mg/mL and 2 mg/mL, respectively. Interestingly, both venoms could be effectively neutralized by anti-disintegrin (Figure 7A,B) similar to Antivenin Polyvalent (Wyeth) antivenom. Pre-immune rabbit serum was used as a negative control and did not neutralize the crude *C. o. helleri* or *C. atrox* proteolytic activity. To test for anti-disintegrin neutralization against additional crude venoms, we used nine other venoms from *C. atrox* (6), *C. o. helleri* (2), and *C. s. scutulatus* (1) in an in vitro gelatinase assay. The MGD was determined for each venom. All the *C. atrox* venoms had an MGD ranging from 0.125 to 2 mg/mL. The two *C. o. helleri* had an MGD of 0.125 and 2 mg/mL, respectively. The only *C. s. scutulatus* venom had an MGD of 0.5 mg/mL. In the gelatinase assay, each venom was incubated with anti-disintegrin and a 2MGD for 30 min at room temperature prior to being applied to the gelatin film. Our results indicated that crude venoms from this study were completely neutralized using anti-disintegrin (Supplemental Figure S3A).

We then sought to determine if anti-disintegrin could neutralize crude venom in a hemorrhagic assay. Crude snake venom from *C. atrox* was used and we first determined the minimal hemorrhagic dose (MHD), which suggested that 2.5 µg of crude venom was required for a 10 mm hemorrhagic spot. The MHD was then used to test anti-disintegrin for neutralization. We incubated our anti-disintegrin with 2MHD prior to injecting sub-dermally in a mouse for the hemorrhagic assay and after the 24 h, the hemorrhagic spot was compared to the MHD and was significantly reduced with ~80% neutralization by anti-disintegrin, compared to serum controls (Figure 7C,D).

## 3. Discussion

Herein, we describe, the isolation of a native disintegrin from *C. atrox* venom (Figure 1) in which the disintegrin activity was neutralized by a novel antibody produced against the disintegrin from the Mojave rattlesnake (Figure 2 and Figure 3). In addition, we isolated PII/PIII SVMPs from *C. o. helleri* (Figure 4), and using the novel disintegrin antibody, were able to neutralize the proteolytic activity in vitro (Figure 5). We also described, to our knowledge, the first use of a novel antibody that targets the disintegrin domain of SVMPs in *Crotalus* crude venom, and is able to neutralize 11 *Crotalus* snake venom’s proteolytic activity in vitro and *C. atrox* hemorrhagic activity in vivo (Figure 6 and Figure 7).

Snakebite envenomation is considered a neglected tropical disease as of 2016 according to WHO (report 2016), resulting in ~81,000 to 138,000 deaths each year [19]. Snake venom contains enzymatic zinc dependent metalloproteinases that degrade the basement membranes, endothelial cells, cause hemorrhaging and activates the coagulation cascade [20,21]. When a substantial amount of venom is injected, the site of envenomation leads to severe tissue necrosis. Most of the patients that are envenomated experience pain, swelling, edema around the injury [5]. Some patients suffer from acute renal failure, anaphylaxis, consumptive coagulopathy, and hypovolemic shock [22], however, this is more prominent in underdeveloped countries where medical care is scarce and snakebite therapy is delayed.

Conventional snakebite envenomation treatment is through the use of commercial antivenoms developed in animals that have been immunized with various snake venoms of different species. However, antivenom is expensive, can cause both acute (urticaria, nausea, vomiting, headache and fever) and delayed (serum sickness) adverse reactions upon administration [23]. Therefore, the necessity to explore alternative strategies for developing treatments against snakebite envenomations is warranted. Indeed, it is conceivable to consider different classes of molecules to meet the deficiency of antivenoms such as synthetic neutralizing molecules, bacterial phages, and mono- and polyclonal antibodies [24]. These molecules can potentially be more efficacious, reduce the cost, and the side effects that current antivenoms encounter.

In this study, the neutralization of disintegrins and SVMPs were tested using polyclonal antibodies against disintegrins to determine if neutralizing the disintegrin domain of SVMPs-PII and PIII will reduce the hemorrhagic activity. Within snake venom, there are two groups of proteins that are known to have the ability to bind to integrins, disintegrins and C-type lectins. Disintegrins can be expressed from specific short-coding mRNAs, which were identified from the venom gland cDNA [25] or from proteolytic processing of larger molecules from the SVMP families PII and PIII, containing disintegrin domains C-terminal to a metalloprotease [26,27]. Therefore, we hypothesized that the disintegrin domain could be used to target PII and PIII-SVMPs. *Crotalus atrox* venom contains mostly SVMPs, which can be as high as 70% of total venom composition relative to other venom family members such as the SVSPs or PLA_2_s [2]. 

Anti-disintegrin was made against r-mojastin 1 that was produced from our lab [17,28]. R-mojastin 1 is a cloned recombinant single chain disintegrin containing an RGD binding motif and codes for 71 amino acids, with 12 cysteines [28]. In this study, we also used a native disintegrin that was purified from the venom of the *C. atrox* (Figure 1). This disintegrin has high amino acid sequence homology with other medium sized disintegrins of the genus *Crotalus.* For example, the disintegrins purified was crotatroxin, which has the same amino acid sequence as hordistatin from *C. horridus* and mojastin from *C. s. scutalatus* [2]. The tri-peptide (RGD) in disintegrins blocks the biological functions of integrins and inhibits platelet aggregation [29,30,31]. A native disintegrin was first tested for platelet inhibition using a platelet aggregometer. At a concentration of 1 mg/mL, only 30% aggregation (70% inhibition) was observed typical of and in the range for well characterized disintegrins. The disintegrin/anti-distintegrin polyclonal antibody mixture was able to neutralize the disintegrin activity, restoring normal platelet aggregation similar to PBS controls (Figure 2A,B). There has been other disintegrins such as, viridistatin, mojastin, and crotostatin which have the same platelet aggregation activities [28,32,33,34]. These results indicate that anti-disintegrin is able to neutralize the native disintegrin activity in vitro.

The native disintegrin D1 purified from the venom of the *C. atrox* were tested for activity using a wound-healing assay to monitor migration of SK-MEL-28 cells. The survival of cells depends on cell-matrix adhesion and cell–cell interaction [35]. Integrin receptors mediate cell adhesion within the extracellular matrix [35]. Cell migration plays an essential role in wound healing and repair; however deregulation can lead to tumor metastasis and inflammatory diseases [36]. D1 was able to inhibit cell migration (Figure 3A,B). D1 was then incubated with anti-disintegrin polyclonal antibody and cell migration was monitored. We found that anti-disintegrin was able to neutralize D1 allowing for complete cell migration (Figure 3C,D). Interestingly, we did not observe any impact of native disintegrin to inhibit cell proliferation (Supplemental Figure S1). It is not unreasonable to assume that anti-disintegrins may target the RGD tri-peptide that binds to the α_IIb_β_3_ integrin, however further analysis using peptide epitope mapping is needed to confirm this.

Certainly, anti-disintegrins have been able to detect the presence of native disintegrins in Mojave rattlesnake venom through immunoblot and enzyme-linked immunosorbent assay (ELISA) [17]. Anti-disintegrins identified SVMPs by showing an increase in the binding ability at the molecular weight range that corresponds with SVMPs [17]. In this study, anti-disintegrin was utilized for the detection of disintegrins and SVMPs from fractionated venom of a Southern Pacific rattlesnake using an immunoblot, as high binding intensity of immuno-reactive bands was observed at the molecular weight of SVMPs for fractions 1,7, and 8 (Figure 4D). N-terminal sequencing revealed the identity of fraction 7 as a metalloproteinease (Supplemental Figure S2A). Although r-mojastin 1 was cloned from a Mojave rattlesnake venom gland, anti-disintegrin is also capable of identifying disintegrins and SVMPs domains from venom of different *Crotalus* species (Supplemental Figure S2C,D).

SVMPs have shown to contribute to a major portion of hemorrhagic activity upon envenomation. The RGD motif found in a significant amount of disintegrins is derived from the subgroup type PIII-SVMPs that contain the ECD amino acid sequence [30,37]. It is very likely that anti-disintegrin is capable of recognizing the disintegrin domain due to the homology in the P-II and P-III disintegrins domains [38]. Anti-disintegrin was used to neutralize the activity of a purified SVMP P-II in a gelatinase assay (Figure 5) and neutralization was observed. These data suggest that anti-disintegrin is possibly inhibiting the activity of the venom by just targeting the disintegrin domain of the protein. Indeed, proteomic analysis revealed the abundance of SVMPs as one of the top protein families in the venom of *C. o. helleri* and *C. atrox* venoms (Figure 6D,E), and further suggest that neutralization of proteolytic activity from crude venom can be achieved using anti-disintegrin.

To confirm this, neutralization of gelatinase activity by anti-disintegrin was tested on different snake venoms of the same species (Supplemental Figure S3B). Anti-disintegrin was capable of neutralizing 3 species from 11 snake venoms used in our study. This was of no surprise since it is well known that venom variations within snakes of the same species can exist [39,40,41]. It has been reported that the neutralization of antivenom is widely based on the intraspecific and interspecific variation within snake venom [42]. Although 100% neutralization of gelatinase activity was achieved by anti-disintegrin, 80% neutralization activity was observed (Figure 7) on hemorrhagic activity using *C. atrox* venom. Since 30–70% of the total venom proteome of a *C. atrox* is made up of two major proteins which are SVMPSs and SVSPs [2], targeting SVMPs and SVSPs can be a novel strategy for developing effective snake envenomation antidotes. Anti-disintegrin may be binding to the disintegrin domains of the SVMPs affecting their folding/structure or substrate binding, leading to the inhibition of their proteolytic activities. Previous study suggests that the C-terminus section of the disintegrin like domain is crucial for binding collagen (one of many major components that comprises basement membranes of cells), and for the hemorrhagic and proteolytic activities of P-III metalloproteinases [43], and inhibition of these activities was achieved by two different monoclonal antibodies targeted to the disintegrin-like domain at the C-terminus [44,45]. Other studies using human monoclonal antibodies have demonstrated neutralization of P-III SVMPs by binding to epitopes near the active site, thus making the SVMPs less efficient in their activities [46]. Inasmuch as intraspecies variation increases the complexity, the different isoforms of SVMPs are highly variable. Sequence alignment of 5 sequenced SVMP disintegrin domains from *C. atrox* showed 11–37% sequence identity for RGD containing disintegrins compared to other disintegrin domains from other PII-SVMPs (Supplemental Figure S3B). This suggest that anti-disintegrin may be able cross-react with the variable SVMP isoforms and neutralize crude venom. Certainly, using a pool of disintegrins for future therapeutic antibody development could also be beneficial. This study signifies a strong first-step for single target neutralization for SVMPs, the capacity of neutralization was limited since this was serum and not purified IgG. It is reasonable to assume a purified anti-disintegrin IgG would be capable of achieving a higher degree of neutralization. To fully test the capabilities of the anti-disintegrin, future studies should include lethality testing in mice to determine whether or not the anti-disintegrin have an effect on the lethal dose of venoms. Besides the possible therapeutic for the development of an effective antivenom, anti-disintegrin could potentially be used as diagnostic tools and used in affinity chromatography to purify metalloproteinase molecules from crude venom and to screen/identify metalloproteinase from other snake venoms.

## 4. Conclusions

In summary, our anti-disintegrin is capable of neutralizing native disintegrin activities. Anti-disintegrin is made against a disintegrin molecule but can neutralize the catalytic site that causes hemorrhaging of SVMPs through an allosteric mechanism as displayed in other enzymes [47,48]. Anti-disintegrin was also capable of neutralizing crude snake venom from snakes of the same species in vitro. Targeting single molecules that can inhibit the whole envenomation effect can open new avenues towards developing novel and improved therapeutics, but also in identifying novel cellular targets of venom toxins providing the foundation for designing innovative therapeutics as well as diagnostic tools for treatment of other diseases.

## 5. Materials and Methods

### 5.1. Collection of Crude Venoms

Snakes were housed at the National Natural Toxins Research Center at Texas A&M University-Kingsville in compliance with IACUC (Approval # 2018-11-09-A3). Venom was extracted by allowing the snakes to bite into a para-film stretched over a disposable plastic cup. The venom sample was centrifuged (500× *g* for 10 min), and filtered through 0.45 µm filter. The venoms were centrifuged for 5 min at 23 °C at 12,800× *g* to remove cellular debris. The venom supernatant was then transferred to vials with the proper labels and stored individually at −80 °C until lyophilized.

### 5.2. Venoms/Anti-Disintegrin

Eleven individual venoms from the species *C. atrox*, *C. o. helleri* and *C. s. scutulatus* from Texas, Arizona, and California were used to evaluate the validity of the anti-disintegrin polyclonal antibodies. Species vial and avid numbers were: *C. atrox* (Luke), na, *C. atrox* (977), 065-115-084, *C. atrox* (53), 010-287-337, *C. atrox* (54), 010-323-805, *C. atrox* (313), 011-513-316, *C. atrox* (312), 011-107-810, *C. atrox* (68), 005-553-539, *C. atrox* (143), 011-052-315, *C. o. helleri* (792), 046-536-058, *C. o. helleri* (680), 058-359-257, *C. s. scutulatus* (931), 065-365-053. Location data is list in Supplemental Figure S3B. It should be noted that the novel antibody described herein is a rabbit polyclonal antibody against R-mojastin1 of the disintegrin molecule cloned from the viper venom gland of *C. s scutulatus*. The serum-containing polyclonal antibody used in this study, (anti-disintegrin) against the mojastin disintegrins was previously characterized in [17] and is a rabbit polyclonal antibody against R-mojastin1 of the disintegrin molecule cloned from the viper venom gland of *C. s scutulatus*. All antibody incubations were premixed with crude venom, fractions or disintegrins at equal volumes (1:1) and carried out at 37 °C for 30 min unless otherwise noted.

### 5.3. Purification of Native Disintegrins by C18-Reverse Phase Chromatography

Ten milligrams of lyophilized crude *C. atrox* venom was reconstituted in 200 µL of 0.1% trifluoroacetic acid (TFA; solution A) and filtered through a 0.45 µm filter. The venoms were then fractionated by reverse phase chromatography using a Higgins Analytical (Mountain View, CA, USA) PROTO 300 C18 (250 × 4.6 mm, 5 µm) column. Fractions were eluted using a 0.1% TFA and 80% acetonitrile (ACN) in 0.1% TFA (solution B) gradient over 60 min, with a flow rate of 1 mL/min. A Waters (Milford, MA, USA) 2487 Dual λ Absorbance detector was used to monitor absorbance at 280 nm. Fractions were stored at −80 °C. Protein concentrations were determined by standard methods at 280 nm using an extinction coefficient of 1 [49,50].

### 5.4. SDS Polyacrylamide Gel Electrophoresis and Immunoblot Analysis

Snake venom or venom fractions were subjected to electrophoresis by using a pre-cast 4–20% Bis-Tri gel in an Xcell SureLock Mini-Cell (Invitrogen Life Technologies, Waltham, MA, USA). A total of 25 µg of crude venom of individual snakes or 5ug of venom fractions were separated on a non-reduced 4–20% Tricine Gel for 95 min at a 100 V using an XCell SureLock^®^ Mini-Cell system (Invitrogen Life Technologies, Waltham, MA, USA). Current was moderated using a Bio-Rad (Hercules, CA, USA) PowerPack power supply. Gels were stained with 50 mL SimplyBlue SafeStain (Invitrogen Life Technologies, Waltham, MA, USA) for 24 h and distained overnight with 18 mega Ohm water. SeeBlue Plus2 markers, ranging from 3 to 210 kDa, were used as standards. For immunoblots, proteins were transferred onto a 0.2 µm Immobilon^®^ membrane (EMD Millipore, Burlington, MA, USA) using a Trans Blot SD system (Bio-Rad, Hercules, CA, USA) at 100 mA for 1.5 h and allowed to set overnight. Blocking was performed in 5% non-fat dry milk/TBST (0.05% Tween-20, 8 mM Tris-Base, 25 mM Tris⋅HCl, and 154 mM NaCl). Primary anti-disintegrin (1:1000 dilution) was incubated with the membranes in 5% (wt/vol) Bovine Serum Albumin (BSA) or 5% non-fat dry milk in TBST overnight at 4 °C and washes were done with TBST. Secondary antibodies (EMD Millipore, Burlington, MA, USA) conjugated to horseradish peroxidase were incubated with the membrane at (1:10,000 dilution) for 1 h at RT, followed by washing with TBST. Membranes were treated with enhanced chemiluminescence (ECL) substrate and visualization was done using Li-COR (Lincoln, NE, USA) C-DiGit^®^ chemiluminescence imaging Blot Scanner.

### 5.5. N-Terminal Sequencing

For the N-terminal sequencing, fractions were transferred from an SDS-PAGE gel onto a 0.2 µm Immobilon^®^ membrane (EMD Millipore, Burlington, MA, USA) using a Semi-Dry Transblot Cell (Bio-Rad, Hercules, CA, USA) at 100 mV for 1.5 h. The membrane was stained with Coomassie R-250 for 5 min. The sample membrane was processed for N-terminal amino acid sequencing using Edman degradation method in a PPSQ-33B protein sequencer (Shimadzu, Japan). The identity of the primary sequence was evaluated using Basic Local Alignment Search Tool-Protein BLAST (BLAST—http://blast.ncbi.nlm.nih.gov/Blast.cgi (accessed on 29 March 2021)).

### 5.6. Platelet Aggregation

Four hundred and fifty microliters of 10% citrated whole human blood were incubated at 37 °C at least 5 min prior to use with equal amounts of 0.15 M sodium chloride. A total of 50 μL of disintegrin was incubated with 50 μL of anti-disintegrin at 37 °C for 30 min. As controls, 50 μL of disintegrin alone (3.5 mg/mL) was incubated with 1× PBS and 50 μL of anti-disintegrin was incubated with 1× PBS at 37 °C for 30 min. A 1× PBS was also used as a PBS control. A total of 10 μL of these samples were incubated with blood samples in a Chrono-log Whole Blood Aggregometer (Havertown, PA, USA) at 37 °C for 2 min. Platelet aggregation was initiated by adding 10 µL of ADP (10 mM). Percent of platelet aggregation was calculated using the following equation:

(E/C) × 100(1)

where C is the units of platelet aggregation (ohms) for the PBS control, and E is the unit of platelet aggregation (ohms) for the experimental fraction. Data were collected by AGGRO/LINK v.5.2.4 provided by ChronoLog Corporation (Havertown, PA, USA on a Dell computer and analyzed by Microsoft Excel for Mac 2019 v.16.43. This assay was repeated three times. The significance was analyzed by the student’s *t*-test, and level of significance was at *p* < 0.05.

### 5.7. Cell Migration Assay

Human melanoma cells, SK-Mel-28 were seeded (5.0 × 10^5^ cells/mL), on a 24 well (35 mm in diameter) microtiter plate containing an insert. After 16 h, the confluent monolayer was observed and the insert was removed leaving a “scratched” (4.0 mm) at the midline of the well. The detached cells were washed away and 1 mL of Modified Eagles Medium (MEM) media was added. The cells received 10 μL of 0.02 M PBS, pH 7.4, 10 μg of D1 or D1 with anti-disintegrin in the same buffer. The cells were then incubated in a 5% CO_2_ chamber and were removed for microscopy images at 0, 6, 8, 12, 24 h. Percent migration was calculated by the following equation:

[(C − E)/C)] × 100
(2)

where C is the distance of cell edge (mm) at zero time of the control, and E is the distance of cell edge (mm) at the final time.

### 5.8. Cell Proliferation Assay

Cell proliferation of SK-Mel 28 cells following treatment with D1 from *C. atrox* venom was measured using the CellTiter-Blue^®^ cell viability assay (Promega, Fitchburg, WI, USA). Two hundred microliters of [51] cells were cultured on 96-well black with clear flat-bottom microtiter plates at 10^5^ cells/well, in triplicates, and incubated overnight at 37 °C in 5% CO_2_. Twenty microliters of D1 at various concentrations (0–1 mg/mL, initial concentrations) were added to the wells and incubated at 37 °C for 24 h. Afterward, 20 μL of the CellTiter-Blue^®^ reagent were then added into each well, and cultured for an additional 4 h. Following the incubation, the fluorescence was recorded at 540/590 nm using a Fluoroskan Ascent FL reader (Thermo Fisher Scientific, Vantaa, Finland). The negative control (control group) was cells treated with PBS buffer, pH 7.4, while the positive control was cells treated with Triton X-100 0.01%. The percentage of inhibition of cell proliferation was calculated relative to the negative control, which was defined as 100% proliferation: inhibition of cell proliferation (%) = (1 − fluorescence of the experimental group/fluorescence of the control group) × 100.

### 5.9. SVMP Purification by Cation Exchange Chromatography

*C. o. helleri* (Vial 792) venom sample (30 mg) was dissolved in 20 mM Tris-HCl pH 8.0 buffer, and run into a cationic exchange column (sulfopropyl waters protein pak 7.5 × 75 mm-10 μm, Milford, MA, USA) equilibrated with 20 mM Tris-HCl, pH 8.0 buffer at a 1 mL/min flow rate. The eluting buffer was linearly integrated from 0% to 100% using a 20 mM Tris-HCl, pH 8.0 buffer containing 0.5 M NaCl. The proteins were eluted at a 1 mL/min flow rate over 90 min using a Waters 1525 binary High Performance Liquid Chromatography (HPLC) system (Milford, MI, USA). A Waters 2487 dual λ absorbance detector (Milford, MI, USA) to monitor absorbance at 280 nm, and Waters (Milford, MA, USA) Breeze software were used to control the HPLC system and save the data. The active fractions were pooled, lyophilized, and stored at −20 °C until used.

### 5.10. Gelatinase Assay

We modified the approach of [52] as described by [53] to test each pooled venom for gelatinase activity. As gelatin is a denatured form of collagen, this assay measures proteolytic (i.e., proteinase) activity; metalloproteinases are the main enzymes responsible for extracellular matrix protein degradation. Twenty microliters of crude venom, venom fractions, 2 mM EDTA, 2 mM PMSF or anti-disintegrin mixtures were placed on a Kodak (Rochester, NY, USA) X-OMAT scientific imaging film with a gelatin coating. The film was incubated at 37 °C for 4 h and then washed with distilled water. Hydrolysis of the gelatin coat (i.e., a clearing of the gelatin on the film) was recorded as a positive result and indicated proteolytic activity; lack of hydrolysis was recorded as a negative result. The minimal amount of venom that causes a clear zone on a Kodak X-OMAT scientific imaging film is defined as the minimal gelatinase dose (MGD). Two times the MGD (2MGD) were incubated 1:1 with venom fractions, 2 mM EDTA, 2 mM PMSF or anti-disintegrin mixtures. This assay was repeated three times.

### 5.11. Proteomic Analysis

Proteins were denatured in 0.1% RapiGest (Waters, Milford, MA, USA) and reduced with 5 mM dithiothreitol for 30 min at 50 °C. Proteins were alkylated in 15 mM iodoacetamide for 1 h in the dark at room temperature and then digested with proteomics grade trypsin at a 1:100 ratio overnight at 37 °C. The pH was adjusted below 3 and the sample was incubated for 45 min at 37 °C. The sample was centrifuged at 16,000× *g* to remove RapiGest. The supernatant was collected. The peptides were dissolved in 5 μL of 0.25% formic acid (FA) with 3% ACN and injected into an Easy-nLC 1000 (Thermo Fisher Scientific, USA). Peptides were separated on a 45 cm in-house packed column (360 μm OD × 75 μm ID) containing C18 resin (2.2 μm, 100 A, (Bischoff Chromatography, Leonberg, Germany) with a column heater (Analytical Sales and Services, Flanders, NJ, USA) set at 50 °C. The mobile phase buffer consisted of 0.1% FA in ultra-pure water (buffer A) with an eluting buffer of 0.1% FA in 80% ACN (buffer B) run over a linear 60 min (method comparisons) gradient of 5–30% buffer B at a flow rate of 250 nL/min. The Easy-nLC 1000 was coupled online with an LTQ-Orbitrap Velos Pro mass spectrometer (Thermo Fisher Scientific, USA). The mass spectrometer was operated in the data-dependent mode in which a full MS scan (from *m*/*z* 350-1500 with the resolution of 30,000 at *m*/*z* 400) was followed by the 10 most intense ions being subjected to collision-induced dissociation (CID) fragmentation. CID fragmentation was performed and acquired in the linear ion trap (normalized collision energy (NCE) 30%, AGC 3e4, max injection time 100 ms, isolation window 3 *m*/*z*, and dynamic exclusion 60 s). The raw file was searched directly against all of the *Crotalus* proteins available in Uniprot (downloaded July, 2018) with a 1% False Discovery Rate (FDR) cutoff at the protein, peptide, and modification levels. The first peptide precursor mass tolerance was set at 10 ppm, and MS/MS tolerance was set at 0.6 Da. Search criteria included a static carbamidomethylation of cysteines and variable modifications of (1) oxidation on methionine residues, (2) acetylation at the N-terminus of proteins.

### 5.12. Hemorrhagic Analysis

To determine the hemorrhagic activity of *C. atrox*, 100 μL of crude venom or venom/anti-disintegrin were subcutaneously injected (s.c.) into the abdominal space of a BALB/c mouse. Each sample was done in triplicate with a single mouse used per sample injected. The mice were sacrificed after 24 h and the skins were removed. Hemorrhagic activity was determined by the presence of a hemorrhagic spot on the skin. This activity was compared with minimum hemorrhagic dose (MHD: 2.5 μg) of *C. atrox* crude venom.

### 5.13. Ethical Procedures

All animal handling procedures were approved by the Texas A&M University-Kingsville Institute of Animal Care and Use Committee (IACUC approval (9 November-2018) #s 2018-11-09-A3).

## Data Availability

Not applicable.

## Supplementary Materials

The following are available online at https://www.mdpi.com/article/10.3390/toxins13040254/s1, Figure S1. Cell proliferation assay. Figure S2. (A) N-terminal sequence of SVMP from *C. o. helleri*. The identity of the primary sequence was determined using Basic Local Alignment Search Tool (BLAST http://blast.ncbi.nlm.nih.gov/Blast.cgi (accessed on 29 March 2021)). (B) HPLC profiles of crude venoms. A total of 500 µg was injected into a Reverse Phase C18 column as described in the Materials and Methods. (C) Western Blot of crude venoms probed with anti-disintegrin. Five micograms of crude venom was separated on SDS-PAGE and were transferred to an Immobilon^®^ membrane (Millipore) using BioRad Trans-Blot SD Semi-Dry Transfer Cell at 100V for 1 h and then leftover night. M: Seeblue^®^ Plus 2 prestained standard (1X), lanes 1: *C. atrox* (Luke), Lane 2: *C. atrox* (977), Lane 3: *C. atrox* (53), Lane 4: *C. atrox* (54), Lane 5: *C. atrox* (313), Lane 6: *C. atrox* (312). (D) Western Blot crude venoms probed with anti-disintegrin M: Seeblue^®^ Plus 2 prestained standard (1X), lanes 1: *C. atrox* (68), Lane 2: *C. atrox* (143), Lane 3: *C. o. helleri* (792), Lane 4: *C. o. helleri* (680), Lane 5: *C. s. scutulatus* (931). Figure S3. (A) Table of snake, identification, location and MGD of venom tested using anti-disintegrin. (B) Sequence alignment of *C. atrox* SVMP PII. The alignment was generated with the ClustalW2 multiple sequence alignment program (https://www.ebi.ac.uk/Tools/msa/clustalw2/ (accessed on 29 March 2021)).
