# Supplementary material for: Examination of the Efficacy and Cross-Reactivity of a Novel Polyclonal Antibody Targeting the Disintegrin Domain in SVMPs to Neutralize Snake Venom
Source: Toxins (Basel). 2021 Mar 31;13(4):254. doi: 10.3390/toxins13040254 (PMC8066378; doi:10.3390/toxins13040254)
Supplement: Supplementary file 1 [file toxins-13-00254-s001.pdf]

Supplementary Materials: Examination of the Efficacy and Cross-Reactivity of a Novel Polyclonal Antibody Targeting the Disintegrin Domain in SVMPs to Neutralize Snake Venom

Shelby S. Szteiter, Ilse N. Diego, Jonathan Ortegon, Eliana Salinas, Abcde Cirilo, Armando Reyes, Oscar Sanchez, Montamas Suntravat, Emelyn Salazar, Elda E. Sánchez and Jacob A. Galan

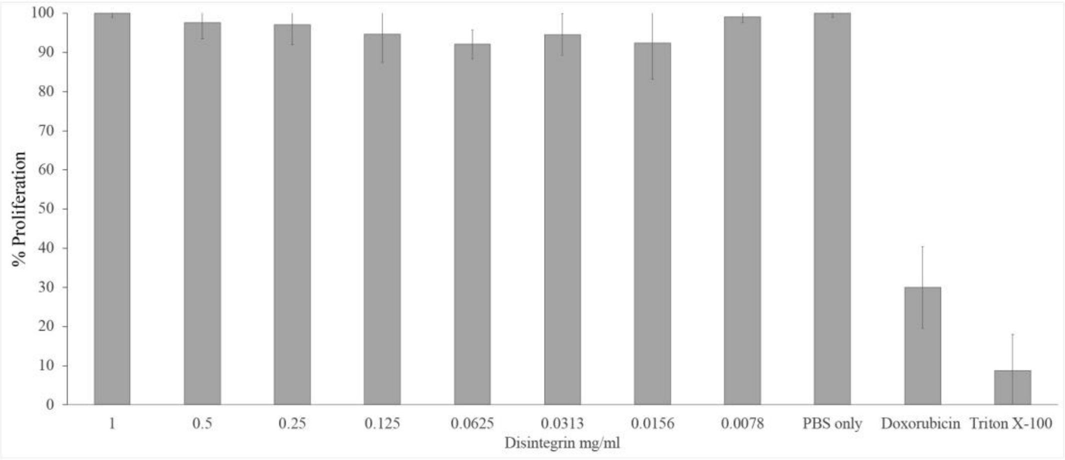

Figure S1. Cell proliferation assay.

A.

Name	N-terminal sequences	Identification/comments
SVMP	GPGIGFNNVDAGLG	Metalloproteinase-disintegrin Q9DGB9.1

B.

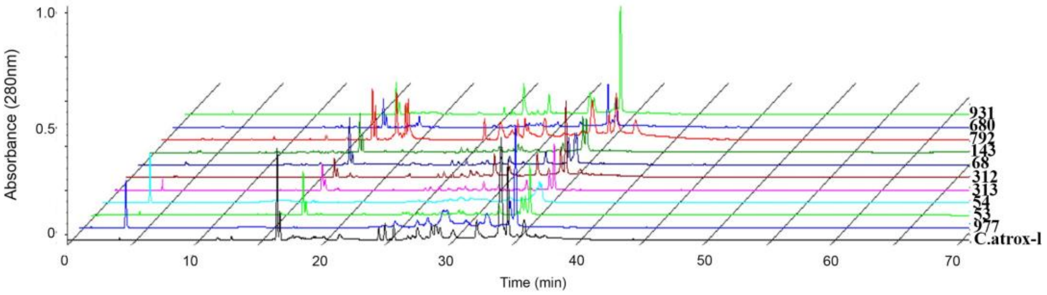

C.

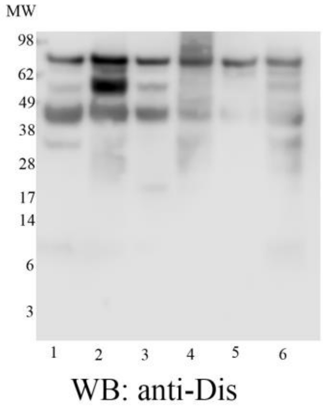

D.

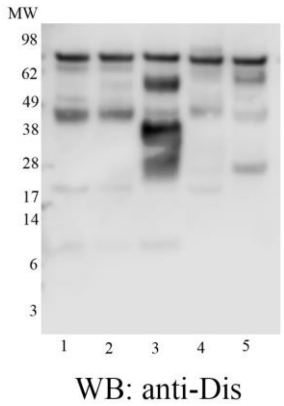

Figure S2. (**A**) N-terminal sequence of SVMP from C. o. helleri. The identity of the primary sequence was determined using Basic Local Alignment Search Tool (BLAST http://blast.ncbi.nlm.nih.gov/Blast.cgi). (**B**) HPLC profiles of crude venoms. A total of 500 µg was injected into a Reverse Phase C18 column as described in the Materials and Methods. (**C**) Western Blot of crude venoms probed with anti-disintegrin. Five micorgrams of crude venom was separated on SDS-PAGE and were transferred to an Immobilon® membrane (Millipore) using BioRad Trans-Blot SD Semi-Dry Transfer Cell at 100 V for 1 h and then leftover night. M: Seeblue® Plus 2 prestained standard (1X), lanes 1: C. atrox (Luke), Lane 2: C. atrox (977), Lane 3: C. atrox (53), Lane 4: C. atrox (54), Lane 5: C. atrox (313), Lane 6: C. atrox (312). (D) Western Blot crude venoms probed with anti-disintegrin M: Seeblue® Plus 2 prestained standard (1X), lanes 1: C. atrox (68), Lane 2: C. atrox (143), Lane 3: C. helleri (792), Lane 4: C. helleri (680), Lane 5: C. s. scutulatus (931).

A.

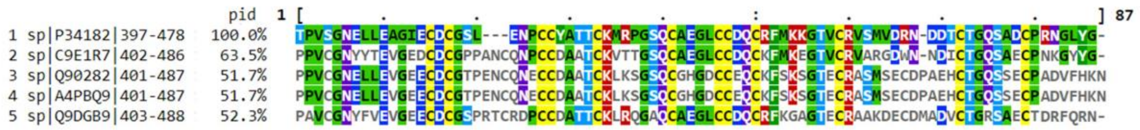

B.

Species	Vial #	Location	MGD (mg/mL)	Neutralized
C. atrox	Luke	Kleberg Co, TX	0.5	+
C. atrox	977	Kleberg Co, TX	2	+
C. atrox	53	San Patricio Co, TX	0.5	+
C. atrox	54	San Patricio Co, TX	1	+
C. atrox	313	Pima Co, AZ	0.25	+
C. atrox	312	Tucson Co, AZ	0.25	+
C. atrox	68	La Salle/ Dimmit Co, TX	0.25	+
C.atrox	143	Culberson Co, TX	0.125	+
C. o. helleri	792	NNTRC Serpentarium	2	+
C. o. helleri	680	San Bernadino, Co. CA	0.125	+
C. s. scutulatus	931	Pima, Co. AZ	0.5	+

Figure S3. (**A**) Sequence alignment of C. atrox SVMP PII. The alignment was generated with the ClustalW2 multiple sequence alignment program (https://www.ebi.ac.uk/Tools/msa/clustalw2/). (**B**) Table of Snake, identification, location and MGD of venom tested using anti-disintegrin.
